# Supplementary material for: Dynamic spatio-temporal patterns of metapopulation occupancy in patchy habitats
Source: R Soc Open Sci. 2021 Jan 13;8(1):201309. doi: 10.1098/rsos.201309 (PMC7890491; doi:10.1098/rsos.201309)
Supplement: Wetlandscape hydrologic model;Texas sensitivity analysis;Comparison between network metrics;Temporal and spatial PDFs for metapopulation occupancy; Zonation in N. Dakota wetlandscape [file rsos201309supp1.docx]

**Supporting Information for:**

**Dynamic Spatiotemporal Patterns of Metapopulation Occupancy**

**in Patchy Habitats**

**List of authors:** L. E. Bertassello^1^, E. Bertuzzo^2^, G. Botter^3^, J. W. Jawitz^4^, A. F. Aubeneau^1^, J. T. Hoverman^5^, A. Rinaldo^3,6^, P. S. C. Rao^1^

^1^ Lyles School of Civil Engineering, Purdue University, West Lafayette, IN 47907-2051, USA

^2^ Dipartimento di Scienze Ambientali, Informatica e Statistica, Università Ca’ Foscari Venezia, 30172 Venezia-Mestre, Italy;

^3^ Department of Civil, Architectural and Environmental Engineering, University of Padua, I-35100 Padua, Italy;

^4^ Soil and Water Sciences Department, University of Florida, Gainesville, FL 32611, USA

^5^ Forestry and Natural Resources, Purdue University, West Lafayette, IN 47907-2051, USA

^6^ Laboratory of Ecohydrology, École Polytechnique Fédérale de Lausanne, 1015 Lausanne, Switzerland;

**Corresponding author:** Leonardo Enrico Bertassello, lbertass@purdue.edu

**SUPPORTING INFORMATION**

**S1: Wetlandscape hydrologic model**

We considered wetlandscapes, populated by distributions of independent GIWs, all share the same rainfall input (P), evapotranspiration loss (PET), and shallow groundwater depth ($z_{gw}$). Over spatial scales considered here (~order of 10^2^ km^2^), spatial variabilities are likely to have smaller impact, relative to their temporal fluctuations. Given these assumptions, we model the temporal dynamics of each wetland comprising the wetlandscape following Bertassello et al. (2019). The water-balance equations for the wetlandscape coupled with shallow groundwater is written as:

$$\frac{dV_{i}}{dt}=PA_{c,i}-A_{i} ET-K_{1}\left[ h_{i}-z_{gw} \right]S_{i} (1S)$$

$$\frac{dV_{gw}}{dt}=R A_{gw}-ET\left( A_{gw}-\sum_{i=1}^{N} A_{i} \right)+K_{1}\left( \sum_{i=1}^{N} [h_{i}-z_{gw}]S_{i} \right)-K_{2}V_{gw} (2S)$$

In Eqs. (1S) and (2S), the subscripts $i$ and $gw$ indicate individual GIWs and shallow groundwater. In particular, Eq. (1S) simulates the daily temporal dynamics of i-wetland volume, while Eq. (2S) simulates the daily temporal dynamics of shallow groundwater. A schematic conceptualization of the model is shown in Figure S1. The first term on the right-hand side of Eqs. (1S) and (2S) represents the daily water inputs due to a sequence of rainfall events. The rainfall input, *P*, in Eq. (1S) is the *actual rainfall*, which occurs over the wetland contributing area, $A_{c,i}$, and is assumed to be collected in the wetland-*i*, while rain falling outside this contributing area is considered to be collected either by another wetland contributing area or by the groundwater contributing area ($A_{gw}$). The recharge term, *R*, in Eq. (2S) is the water input from the root zone to the groundwater due to a sequence of censored rainfall events over $A_{gw}$, corrected by the removal of wetland surface area ($\sum_{i=1}^{N} A_{i}$). Following Laio et al. (2001) and Botter et al. (2007) the functional relation between the rainfall events, *P*, and the recharge, *R,* are represented in terms of a censoring threshold $\delta$, which incorporates the effect of soil-water dynamics and evapotranspiration losses from the root zone: $R=(P-\delta).$

The value of $\delta$ is calculated as a function of rainfall, soil and vegetation properties under the following hypotheses: i) the incoming rainfall is a stationary marked Poisson process with frequency $\lambda_{p}$, and exponentially distributed depths with mean $\alpha_{p}$; and ii) recharge pulses beyond the root zone are triggered by the exceedance of a field capacity $s_{fc}$ in response to precipitation. Under these assumptions the effective censoring threshold, $\delta$, is expressed as (Laio et al., 2001; Porporato et al., 2004; Botter et al., 2007):

$$\delta=\alpha_{p}\ln\frac{\gamma\Gamma(\frac{\gamma}{\phi},\gamma)}{\phi\gamma^{\gamma/\phi}e^{-\gamma}} (3S)$$

where $\phi$ is the aridity index (Budyko, 1974), which is the ratio between the mean potential evapotranspiration (PET) and the mean rainfall $\left\langle P \right\rangle=\alpha_{p}\lambda_{p}$, and $\gamma$ defines the ratio between the soil-water storage capacity $nZr(s_{fc}-s_{w})$ and the mean rainfall depth $\alpha_{p}$, where $n$ is the porosity of the soil, $Zr$ the root zone depth, and $s_{fc}$ and $s_{w}$ representing respectively, the field capacity and the wilting point. In Eq. (3S), $\Gamma(\gamma/\phi,\gamma)$ represents the lower incomplete gamma function of parameters $\gamma/\phi$ and $\gamma$. The values of soil parameters that are used in the wetlandscape simulations are reported in Table A1.

The second term on the right-hand side of Eqs. (1S) and (2S) accounts for the volumetric water loss from the wetland at a temporal-average PET rate, where $A_{i}$ the water surface area of wetland $i$ which changes over time. In Eq. (2S), PET losses are from the whole landscape area that is not inundated.

The third term on the right-hand side of Eqs. (1S) and (2S) describes the rate of exchange between wetlands and shallow groundwater, through wetted area of the pool, $S_{i}$. This process is modeled following a generalized Darcy’s law, where the hydraulic gradient is defined by the difference between wetland pool level, $h_{i}$ and groundwater stage, $z_{gw}$, and $K_{1}$ [T^-1^] is the recession constant of the wetland bottom layer. In Eq. (2S), this term sums the entire water flux exchange between all N wetlands and the shallow groundwater. We assumed that regional differences in $K_{1}$ among wetlandscapes dominates local differences between individual wetlands, and thus each wetlandscape has a distinct $K_{1}$ value that is used for each wetland within a given wetlandscape.

The last term on the right-hand side of Eq. (2S) expresses losses from regional groundwater recession from gravity-driven vertical leakage. Accordingly, $K_{2}$ [T^-1^] is the recession constant of shallow groundwater.


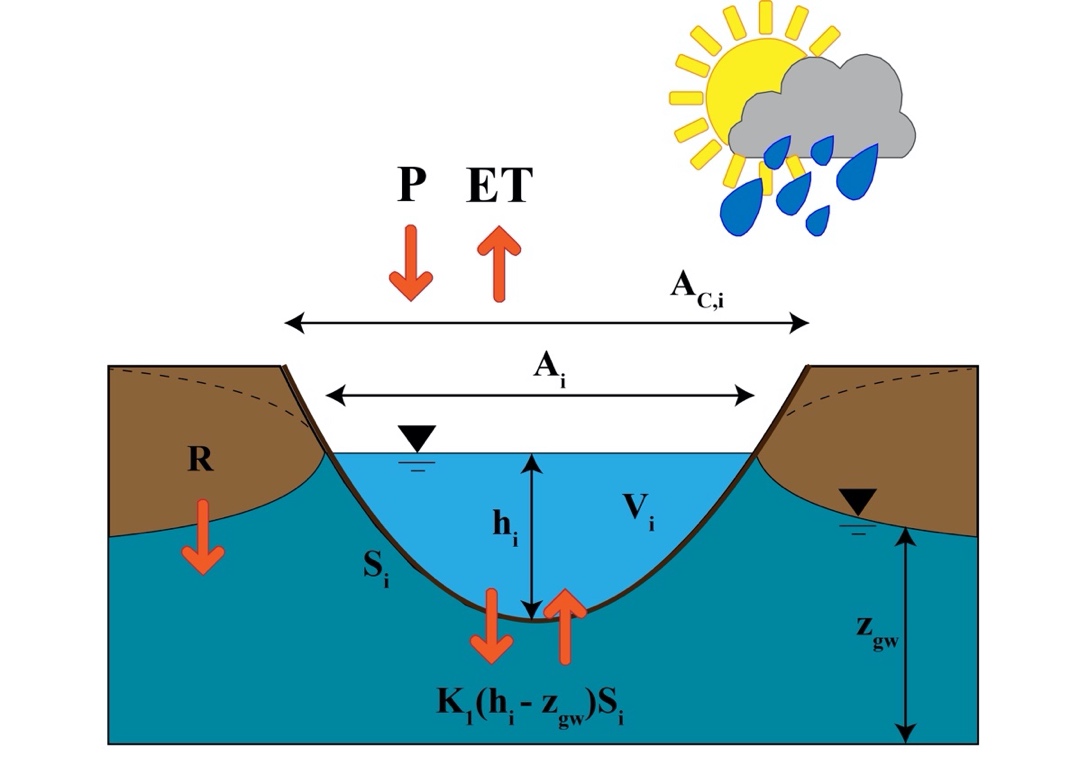


**Figure S1**. Schematic conceptualization of the wetland-groundwater model proposed in Eqs. (1) and (2) for a given wetland i. Rainfall (P), potential evapotranspiration (PET), and recharge (R) represent the impact of hydroclimatic forcing. The term $K_{1}\left( h_{i}-z_{\mathrm{gw}} \right)S_{i}$ represents the exchange of water between the wetland and groundwater over wetted area, $S_{i}$ . The other terms define wetland geometrical attributes. (Adapted from Bertassello et al., 2019).

**S2: Texas sensitivity analysis**

Here, we report the results of sensitivity analysis on survived metapopulation for Texas wetlandscape based on the variation of species traits ($e$,$c$) and hydrological variability ($\alpha$, $\lambda$). The analysis is done for both the static and dynamic SPOM. As it is also shown in Figure 3 of the main text, the dynamic approach is characterized by a larger zone of metapopulation extinction if compared with the static SPOM.

**
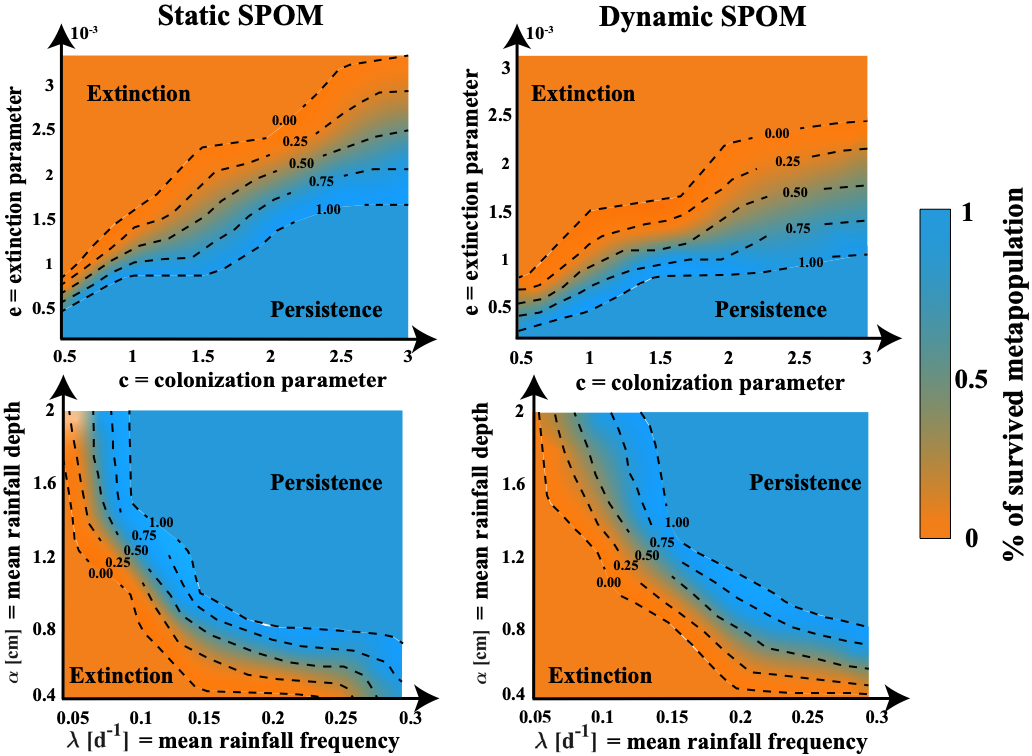
**

**Figure S2**. Sensitivity of static and dynamic SPOM simulations to changes in species traits ($e$,$c$) and hydrological variability ($\alpha$, $\lambda$) on metapopulation persistence wetlandscapes. (A) Results are shown for sensitivity to species traits, $e$ [0.0001;0.0032] and $c$ [0.5;3.0], with fixed ratio $D/\mathrm{NND}=1.5,and h$ydroclimatic parameters ($\alpha=0.90$ cm, $\lambda=0.20$ d^-1^ and $ET=0.50$ cm/d). (B) Sensitivity of static and dynamic SPOM to hydroclimatic parameters $\alpha$ [0.40; 2.00] and $\lambda$ [0.05;0.30], by fixing $ET=0.50$ cm/d, $e=0.0008$, $c=1.5$ and $D/NND=1.5$. For each set of parameter combinations, 100 Monte Carlo simulations were run.

**S3: Comparison between network metrics**

The identification of the key patches in wetlandscape network is of fundamental importance from a landscape management perspective. Node degree and node betweenness give interesting but sometimes different information. As we specified in the main text, node degree is useful to locate the “hubs” (and node clusters) in the network, while node betweenness identifies the “stepping stones” patches that build up an ecological corridor for species dispersal. These two quantities do not always coincide (Figure S3). To understand the degree of correlation between “hubs” and “stepping stones” we ranked the top-20 values for wetland node degree and betweenness. Then we plotted the top-20 hubs against the corresponding node betweenness values, and vice versa. We conclude that, especially in N. Dakota, there is no correlation between the “hubs” and “stepping stones” wetlands, thus in an optic of landscape management it is fundamental to decide what type of intervention we want to pursue. On the other hand, in Texas there is more correlation between these two variables, thus conservation strategies could both enhance wetland clustering and improve the ecological connectivity of the landscape.


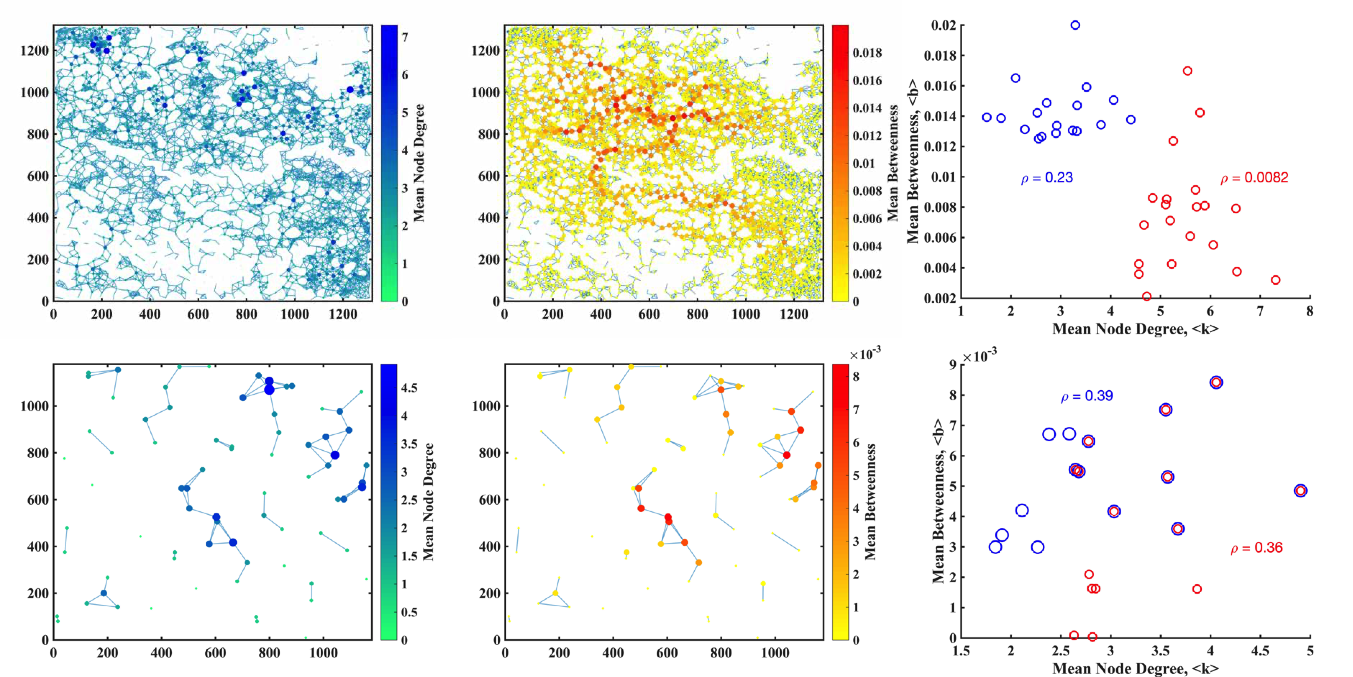


**Figure S3**. Comparison between node degree and node betweenness in N. Dakota (upper panel) and Texas (lower panel). Networks are obtained by fixing the ratio $D/NND=1.5$ for consistency (D = 150 m in N. Dakota and 500 m in Texas).

**S4: Temporal and spatial PDFs for metapopulation occupancy**

There are two important aspects of wetlandscape occupancy that should be considered. First, it is temporally variable because as time goes on we can appreciate the effects of hydrological variability on wetlandscape habitat (Figure 1 of main text). In addition, since the landscape is composed by a large number of patches, the occupancy pattern is also spatially variable. With these premises, we evaluated the probability density functions, PDFs of wetlandscape occupancy of two distinct variables. These two variables are obtained from the binary matrix $\boldsymbol{\Omega}$, whose rows indicate the time series of wetland occupancy, while the columns indicate the occupancy values in each wetland at time $t$.

- $\boldsymbol{\Omega}$ spatially averaged: calculate the average occupancy for each row. The length of this variable is equal to the number of wetlands;
- $\boldsymbol{\Omega}$ temporally averaged: calculate the average occupancy for each column. The length of this variable is equal to the number of time-steps.

Figure S4 shows the PDFs of these two variables. On average, in N. Dakota there are less wetlands occupied by the focal species, while in Texas almost all the wetlands are occupied and they remain in this condition for most of the time. The small peak that is observed for 0.2 < $\boldsymbol{\Omega}$ temporally averaged < 0.4 in Texas, is related to the dry period that occurs in 2003-2005.


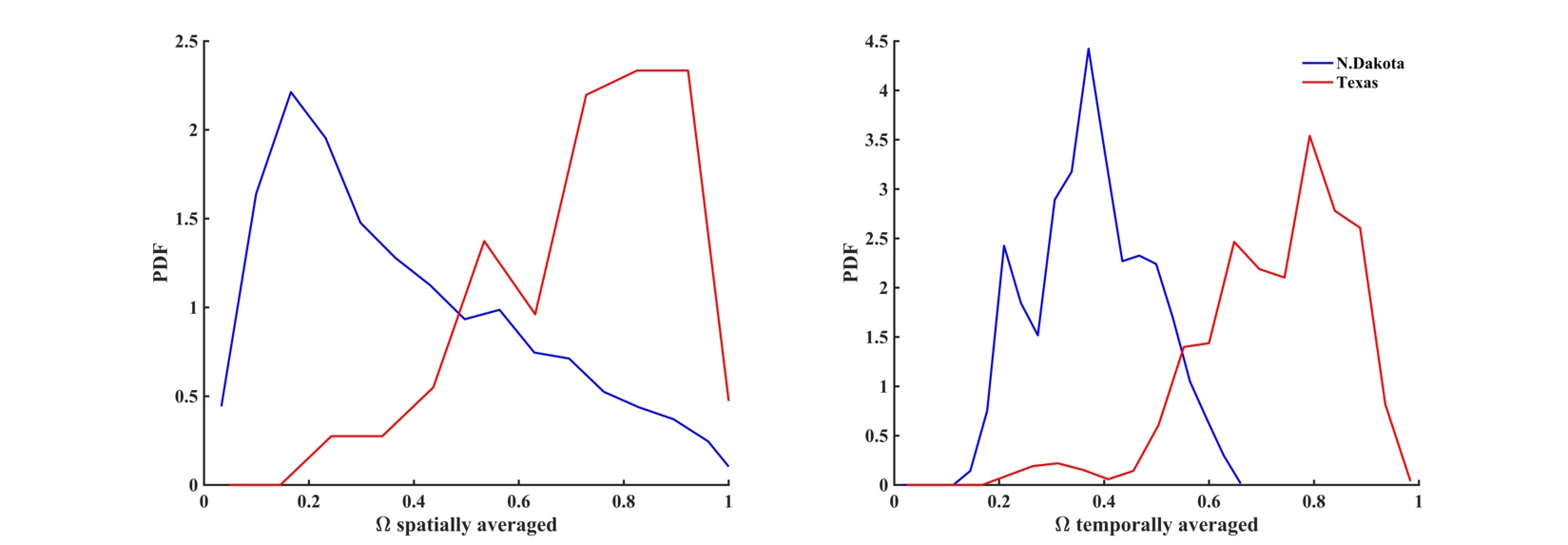


**Figure S4**. Comparison between the PDF of spatially and temporally average value of occupancy in the two wetlandscapes.

To single out the dominant effect between climate and separation distance on metapopulation dynamics, we compared the ratio between the coefficient of variation, CV, of wetlandscape area and nearest neighbor distribution. Each CV is calculated based on the spatial mean and standard deviation at each time step across all the wetlands distributed in the wetlandscape. Larger is the ratio $r_{CV}$ more dominant are the effects of climate variability (manifested in areas variability) over wetlandscape spatial configuration (gap distances). Figure S5 shows that $r_{CV}>1$ in both the landscapes, but especially in N. Dakota, the effects of climate are much larger than the effects of spatial configuration.


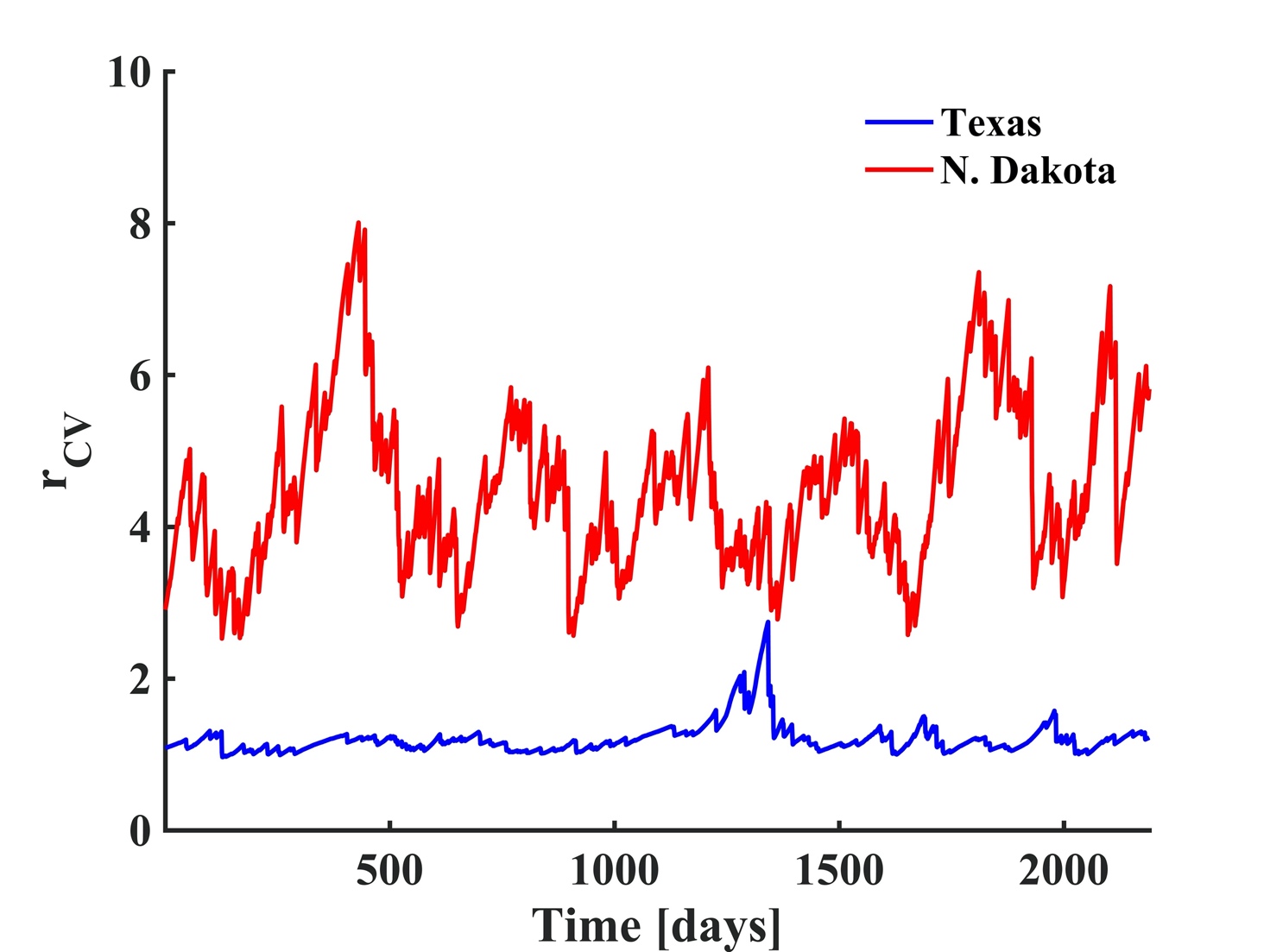


**Figure S5**. Temporal comparison between the ratio between the CV of wetlandscape area and nearest neighbor distance in the two wetlandscapes.

**S5: Zonation in N. Dakota wetlandscape**


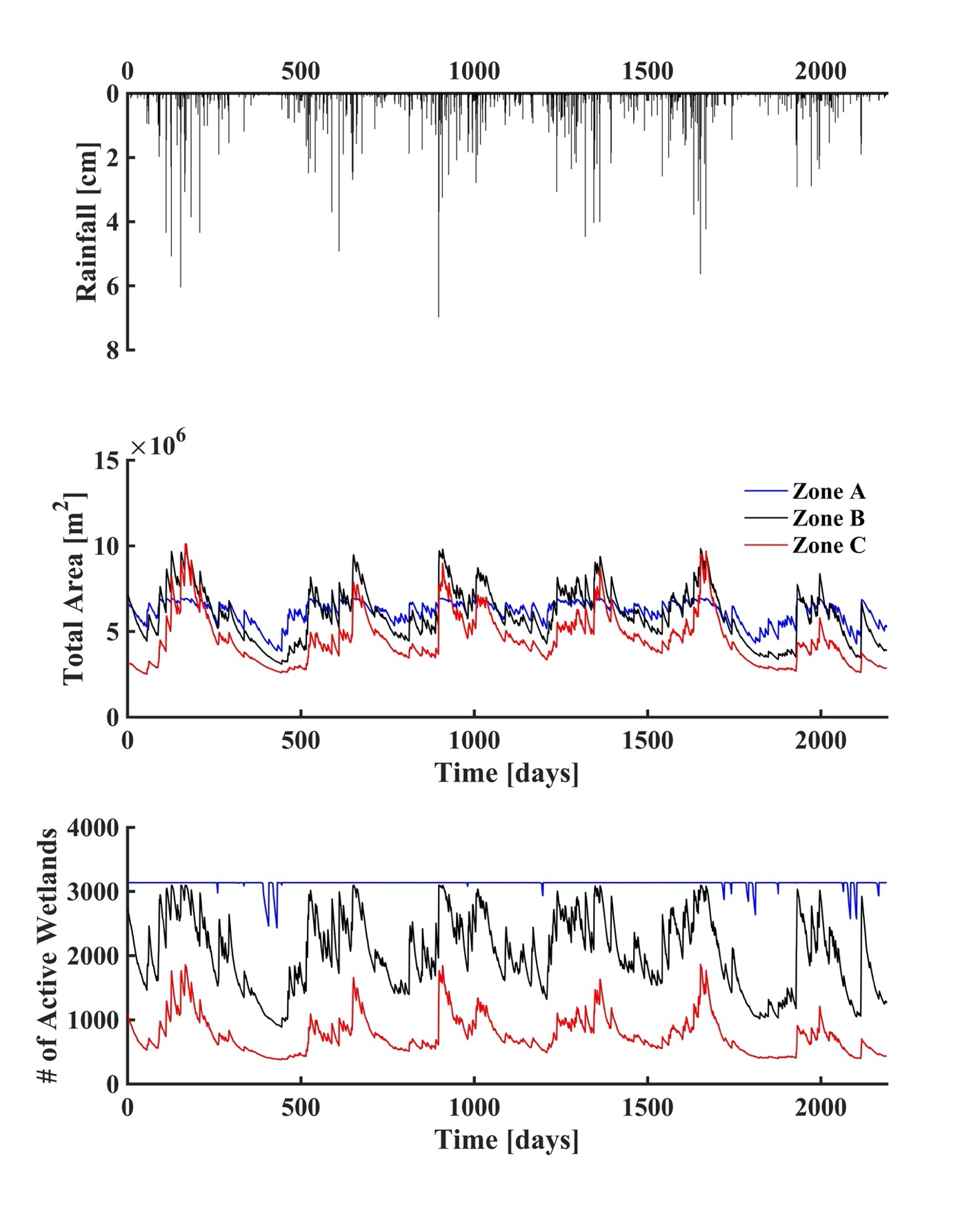


**Figure S6**. Influence of rainfall patterns on the time series of total area of each wetland zone and the number of active wetted zones). Hyetographs (grey histograms) are based on rainfall records.

**REFERENCES**

- Bertassello, L. E., Jawitz, J. W., Aubeneau, A. F., Botter, G., & Rao, P. S. C. (2019). Stochastic dynamics of wetlandscapes: Ecohydrological implications of shifts in hydro-climatic forcing and landscape configuration. Science of the Total Environment, 694, 133765.
- Botter, G., A. Porporato, I. Rodriguez‐Iturbe, and A. Rinaldo (2007), Basin‐scale soil moisture dynamics and the probabilistic characterization of carrier hydrologic flows: Slow, leaching‐prone components of the hydrologic response, Water Resour. Res., 43, W02417, doi:[10.1029/2006WR005043](https://doi.org/10.1029/2006WR005043).
- Budyko, M. I. Climate and life. Academic Press: New York, NY, USA, 1974.
- Laio, F., Porporato, A., Ridolfi, L., & Rodriguez-Iturbe, I. (2001). Plants in water-controlled ecosystems: active role in hydrologic processes and response to water stress: II. Probabilistic soil moisture dynamics. Advances in Water Resources, 24(7), 707-723.
- Porporato, A., Daly, E., & Rodriguez-Iturbe, I. (2004). Soil water balance and ecosystem response to climate change. The American Naturalist, 164(5), 625-632.
